# Evaluation of the Autonomic Nervous System Using the FAN® Device – Range of Normal and Examples of Abnormal

**DOI:** 10.2174/1874205X00802010012

**Published:** 2008-05-07

**Authors:** S Haegele-Link, D Claus, S Dücker, T Vogt, F Birklein

**Affiliations:** 1 Department of Neurology, Kantonsspital St Gallen, Switzerland; 2 Department of Neurology, Hospital of Darmstadt, Germany; 3 Department of Neurology, University of Mainz, Germany

## Abstract

Different components of the autonomic nervous system may be affected by different disorders to varying degrees. The aim of this study is to report first experiences with a new device (FAN®, Schwarzer, Germany) which measures heart rate variability (HRV), sympathetic skin responses (SSR) and the pulse wave transit time (PTT). We examined 190 healthy volunteers (102 men, 88 women) and in 89 subjects (46 men, 43 women) PTT during VM was investigated. In a subset of 24 subjects PTT was compared to conventional blood pressure recording. Thereafter, normal data were compared to patients with polyneuropathy (PNP) and Parkinson syndromes. All parameters of HRV decreased with age. 6 parameters for HRV at rest, during deep respiration and the valsalva ratio were reclassified into three age categories: under 40 (n=96), 40 – 60 (n=71) and 60 or older (n=23). Applying the lower limits of normal (5%-tile) subjects did not have more than 2 of these 6 parameters in the pathological range PTT reduction during phase IV of the valsalva manoeuvre was greater than 7.7 ms (5%-tile) but not age dependent. Patients with PNP had reduced HRV and SSR, Parkinson patients had more frequently impaired blood pressure regulation according to PTT assessment. Our investigation shows that the FAN® might be useful for clinicians to detect autonomic disorders.

## INTRODUCTION

The autonomic nervous system has three major components: Parasympathetic (mainly vagal), sympathetic noradrenergic (vasoconstrictive) and sympathetic cholinergic (sudomotor). All three components may be affected by different disorders to varying degrees. In orthostatic hypotension, which occurs in Parkinson syndromes, sympathetic adrenergic failure may be predominant [1-3]. In diabetic neuropathies, reduction of the vagally mediated heart rate variability can be one of the first symptoms to appear [4-9] and in metabolic neuropathies, such as Fabry’s disease, sudomotor failure occurs early while noradrenergic function is preserved [10-12].The measurement of heart rate variability (HRV) is clinical routine in most autonomic labs. In contrast to these cardio-vagal investigations, it is much more complicated to get quantitative information about sympathetic nervous system function. The quantitative assessment of sudomotor function often requires a very sophisticated equipment [13]. This is why the sympathetic skin response (SSR), which is a more qualitative approach for the assessment of the sweat gland function, was chosen, as SSR can be obtained using standard electromyographic machines [14]. For quantification of blood pressure responses (e.g during standing up or tilting) beat-to beat blood pressure monitoring is required [15, 16]. Unfortunately such monitors are very expensive limiting their clinical use. The reason we performed this study was to report the clinical application of a new device which permits the recording of HRV, blood pressure response and the assessment of the SSR with the same

hardware. The FAN® (Schwarzer, Germany) records HRV and SSR based on the standard recording techniques and algorithms used already in autonomic function studies [17, 18]. Blood pressure changes, however, are estimated using the pulse transit time (PTT). PTT has been proposed to be a substitute for the direct measurement of blood pressure [19], in particular if not the absolute values but blood pressure changes are to be detected [20, 21]. Since PTT requires only simultaneous recording of standard ECG and a pulse wave using photoplethysmography, it simplifies the measurement of blood pressure responses. Although a first study using the FAN® to assess autonomic function has been published [22] there are no reports about normal values assessed by the FAN®. Since it is generally accepted that autonomic function parameters are significantly related to age [23] we investigated a sufficient number of healthy subjects. Using PTT is new in the routine assessment of autonomic function parameters therefore we compared PTT to standard beat-to-beat blood pressure recordings in a subset of these subjects. In order to estimate the clinical significance we thereafter compared our normative data to patients with suspected autonomic disorders.

## MATERIALS AND METHODOLOGY

### Subjects

Normal subjects were 190 volunteers free of any illnesses affecting the heart and the nervous system. 102 of these subjects were men, 88 women. The mean age was 42.9 years, ranging from 16 to 88. Care was taken that none of the subjects were under any medication directly affecting heart rate (vagally or sympathetically). In this respect, ß-blockers, antidepressants and antihypertonics were the most important to consider. Furthermore, the volunteers were instructed to abstain from smoking and drinking coffee on the day of investigation. In all subjects, HRV was assessed according to the protocol given below. In ten of the subjects (2 men, 8 women, mean age 29.0 years (21-51), HRV tests were repeated on another day in order to estimate test-retest reliability. In 89 normal subjects (46 men, 43 women, mean age 42.8 years, range 21-88), the PTT change during Valsava manoeuvre was assessed. This measurement was performed twice in all 89 subjects. In 24 of these PTT was compared to standard beat-to-beat blood pressure measurement using a blood pressure monitor (Finapres; FMS, Amsterdam, The Netherlands). The sequence of PTT and blood pressure measurement was balanced. In order to assess the discriminating value of our normal data, two groups of patients, in whom autonomic dysfunction was common, were investigated. The first group were 34 patients with sensory-motor polyneuropathy (PNP). PNP was diagnosed clinically and confirmed by nerve conduction studies. The aetiology of PNP was diverse. Main causes were diabetes, alcoholism, paraneoplasia and vitamin B 12 deficiency. The mean age of PNP patients was 55.9 years, ranging from 25 to 80. The second group of patients consisted of 22 patients with Parkinson syndromes. According with clinical criteria, patients were classified as typical and atypical (including suspicious multiple system atrophy). For the purpose of our study, all Parkinson syndrome patients were pooled. The mean age in this patient group (11 men and 11 women) was 67 (range 46-83). For obvious reasons, all patients were treated with anti-Parkinson medication (L-Dopa, dopaminagonists, amantadine) which may have interfered with autonomic function tests. In the same way as with the normal subjects, patients were instructed not to take ß-blockers, antidepressants or antihypertonics before measurement.

### Investigation Protocol

As a prerequisite, patients and normal subjects were investigated during morning hours and laid supine for at least 20 minutes in our temperature controlled lab before starting the investigations. At first, heart rate variation during deep respiration was assessed; thereafter, the valsalva manoeuvre was performed and, finally, the heart rate variability at rest for 2 minutes and the heart rate response to active standing up were assessed.

After finishing the HRV assessment, the blood pressure response to the valsalva manoeuvre was assessed. PTT measurement was performed two times; beat-to-beat blood pressure was assessed on the same occasion.

The sympathetic skin response was assessed in patients at the end of the session. Description of the FAN®:

The FAN® has several major components. The most important one is an interface box for recording of ECG, SSR, breathing frequency and expiratory pressure e.g. during valsalva manoeuvre. ECG, SSR and breathing frequency were digitized with a sampling rate of 500 Hz; the expiratory pressure can be recorded with the limits of 0-100 mm mercury. The interface also offers the opportunity to record one analogue external signal such as the pulse-wave or the blood pressure from an external monitor (digitizing rate 500 Hz). The interface also supplies acoustical (1 kHz, 0-100 dB, duration 10-1000ms) and electrical outputs (square wave pulse, 0-30mA, 0.2 ms duration) for triggering the SSR. The second component is a so-called “feedback box”, which displays a bar moving up and down indicating the selected rhythm of breathing for the subjects. Close to the moving bar the actual breathing is displayed by LEDs moving up and down according to the breast excursions. This allows a feed-back control of breathing. The third component is a Laptop PC to process the signals. Internal software automatically extracts relevant parameters (as described below) but also allows the definition of new parameters and test designs. The last component is a printer to make a hard copy of the results.

Heart rate variability (HRV): Subjects were instructed to perform 6 rhythmic breathing cycles per minute by observing the bar moving up and down on the feed-back box. In order to control breathing, a sensor was attached to the subjects’ chests. If necessary, subjects’ breathing was displayed on the feedback monitor close to the moving bar. Heart rate was recorded by standard ECG electrodes fixed at the extremities. During three minutes of deep respiration, the median value of the maximum differences between the longest and shortest R-R intervals (max-min) and the median ratio of the longest and shortest R-R intervals (max/min) within the 18 (=3 x 6/min) breathing cycles were calculated. In addition, the coefficient of variation (VC) and the root mean square of successive differences (RMSSD) of all R-R intervals were obtained.

The Valsalva manoeuvre (VM) was performed after one minute of sitting. After this time, the subjects were instructed by an acoustic beep to maintain an expiratory pressure of 40 mm mercury for 15 seconds. The 40 mm mercury value was displayed as a bar on the feedback control box and the patient´s expiratory pressure was displayed close beneath. The Valsalva ratio (VR) was calculated on the basis of the longest R-R interval within 30 seconds after the manoeuvre and the shortest R-R interval during or within the first five seconds after the manoeuvre.

HRV at rest was recorded when the subjects lay supine for 5 minutes. Breathing cycles were not feedback controlled during this test. The important variables were VC and RMSSD within these 5 minutes.

Directly following HRV at rest, subjects were instructed by an acoustic beep to stand up and stay upright for one minute. The posture index (PI) was calculated on the basis of the longest R-R interval (20 – 40 heartbeats) and the shortest R-R interval (5- 25 heartbeats) after standing up [17].

### Blood Pressure Regulation During VM

As a surrogate of beat to beat blood pressure, the pulse wave latency (PTT) was employed. The PTT was calculated by measuring the interval between the R wave of the ECG and the onset of the pulse wave, which was recorded on the right earlobe by a photoplethysmograph. The VM causes a 4-tailed blood pressure response. It has been shown that late phase II and phase IV depend on the function of the autonomic and, in particular, sympathetic nervous system. Since late phase II is poorly defined on a time basis, this study focuses on phase IV, the blood pressure overshoot (= PTT reduction in relation to baseline) after the VM. Thus, two parameters were calculated, the mean PTT at rest during 30 seconds of sitting before VM and the mean of the 5 shortest PTTs within 15 seconds after the end of the VM. PTT assessment during VM was performed two times consecutively. In a subset of subjects (n=24) the VM was repeated a third time. Now instead of PTT, beat-to-beat blood pressure was directly recorded by a Finapres® monitor and the signal was imported into the FAN®. Due to the fact that the FAN® allows to connect only one external signal, PTT and blood pressure must be recorded subsequently.

### Sympathetic Skin Response (SSR)

The SSR was described more than 100 years ago. Briefly, SSR mainly depends on the differential activation of sweat glands under emotional control at glabrous skin on palms and soles, and thermoregulatory controlled sweat glands on hairy skin. This differential activation can be recorded as a skin potential. Any arousal activates predominantly “emotional” sweat glands, which causes a change of the skin potential. SSR amplitude in healthy subjects varies substantially on different occasions and the latency of this very slow potential is hard to define. However, it has been shown that SSR can be elicited in any healthy subject [14]. Therefore we abstained from collecting useless normative data and focused solely on the presence of the SSR on patients´ feet. The SSR was elicited by the FAN employing a 100 dB acoustic beep.

### Statistical Analysis

Statistical analysis was performed using the software package “SPSS 10.0 for windows” (SPSS Inc., Chicago, Ill., USA). In order to analyze the dependence of the different parameters (HRV and PWL) on age, height and weight, a logarithmic regression was chosen. Influences of sex or smoking habits were determined employing an univariate ANOVA for each variable. The lower limit of normal values was determined as the 5% percentile. Test-retest reliability was estimated using the Pearson correlation coefficient. Healthy control subjects and patients were compared with an ANCOVA model adjusted for age. Parameters were presented as mean +/- SD, where applicable. Significance was considered if p < 0,05 was reached.

## RESULTS

Normal values and test-retest reliability of standard parameters of heart rate variation (HRV).

All 8 parameters of heart rate variation decreased with age in perfect alignment with a logarithmic curve. This is applicable to the four parameters during two minutes of deep respiration (max-min, max/min, VC, RMSSD), the Valsalva Ratio (VR), the two parameters during 5 minutes at rest (VC, RMSSD) and the posture index.

**Fig. (1):**
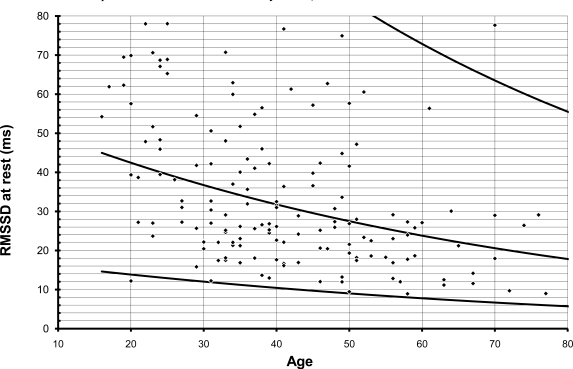
All parameters of heart rate variability decrease logarithmically with age. This graph illustrates distribution of individual RMSSD values. Lines indicate mean, 95% CI and 5% CI of individual values.

**Fig. (2):**
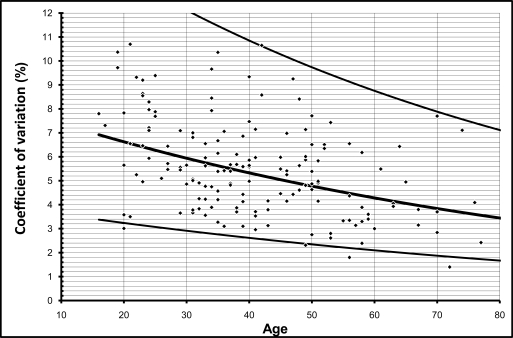
This graph illustrates distribution of individual coefficient of variation values during deep respiration. Lines indicate mean, 95% CI and 5% CI of individual values.

For obvious reasons, max/min during deep respiration,VR and posture index cannot be less than one. Plotting individual values against age revealed that the calculated 5%-tile line did not cross this border in the VR. The lower limit of max/min during deep respiration, however, crossed 1 on the Y-axis below the age of 50, and the lower limit of the posture index was never greater than 1 (see Fig. **1** and **2**). Therefore, these two values were dropped from further analysis.

**Fig. (3):**
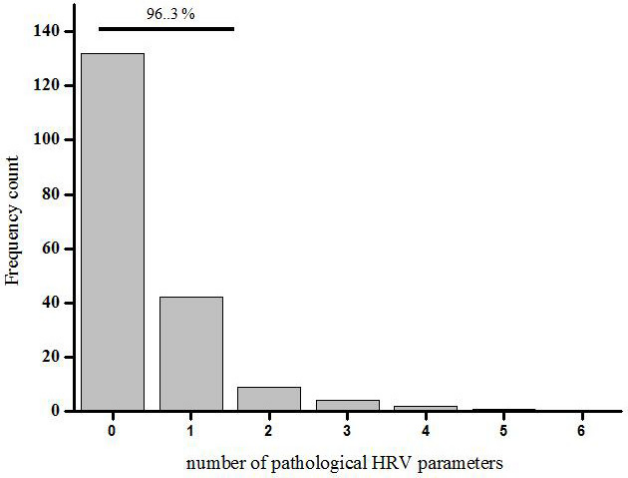
Using the lower limits of normal of 6 HRV parameters normal subjects were reclassified as “normal” or “pathological”. 96% of the normal subjects had 2 or less of 6 parameters in the pathological range.

Subsequently, normal subjects were reclassified into three age groups: below 40 (n=96), 40– 60 (n=71) and 60 or older (n=23). The respective lower limits of normal values of all parameters are shown in Table **1**. Using these limits, the individual values of all subjects were reclassified as “normal” or “pathological”. 95% of normal subjects had 2, or less, of all 8 possible parameters in the pathological range. Recalculation without the min/max ratio during deep respiration and the posture index even leads to the result: 96,3 % had 2, or less, of 6 parameters in the pathological range (Fig. **3**).

In general, neither body weight nor height played significant roles, with one exception: the valsalva ratio increased with increasing height (F=4.86, p<0.03). Sex or smoking habits had no effect on HRV parameters (F< 2 in any case, ns, ANOVA).

Test – retest variability (n=10) was moderate and was maximised for HRV during deep respiration, which was controlled by feedback breathing, and at rest. It was fairly low if more active participation of subjects was required (VR and posture index). For details, see Table **2**.

**Fig. (4):**
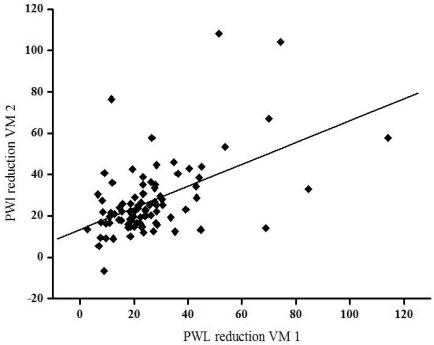
In 89 of normal subjects PTT change during Valsava manoeuvre (VM) was performed twice. Significant test- retest correlation (r=0.52, p<0.001) is shown.

PTT during Valsalva manoeuvre (n=89): The blood pressure overshoot during phase IV of the VM, namely the reduction of PTT in relation to the baseline, was neither significantly affected by age (F=0.64,ns), nor height (F=0.02, ns) nor weight (F=0.98, ns). There was also no significant effect generated by sex (F=0.003) and smoking habits (F=0.95). The 5% -tile of PTT reduction in phase IV was 7.7. This means that a PWL reduction of 7.7, or more, as compared to mean PWL assessed during 30 seconds before the VM should be considered as normal. Test- retest reliability within one experimental session was good. The results of the mean PTT before the VM were significantly correlated in both tests (r=0.83, p<0.001). The results for PTT reduction in phase IV of the VM in both sessions showed less but significant autocorrelation (r=0.52, p<0.001, Fig. **4**). However, test - retest reliability was poor if the test was repeated several weeks later (n=10) and did not reach significance for mean PWL before VM (r=-0.57) and PWL reduction during phase IV (r=0.17).

In the comparative analysis of PTT and beat-to-beat blood pressure (n=24) PTT at rest was 154 +/- 19.2 ms and during phase IV of the VM 144 +/- 17.3 ms (p<0.001). As expected blood pressure increased during phase IV (systolic blood pressure at rest 124 +/-20.5 mmHg, phase IV 150 +/- 29 mmHg, p<0.001; diastolic blood pressure at rest 65 +/-10 mmHg, phase IV 74 +/- 12.7 mmHg, p<0.001; mean blood pressure at rest 95 +/-13.3 mmHg, phase IV 112 +/- 19.7 mmHg, p<0.001) while heart rate slightly but significantly increased (at rest 69 +/- 8.6 min-1; phase IV 72 +/-9.5 min-1, p<0.001).

Correlation analysis revealed that before VM systolic blood pressure at rest (r=-0.59, p<0.005) and mean blood pressure (r=-0.50, p<0.02) were both negatively correlated to PTT. After VM (phase IV) only the heart rate was positively correlated to PTT (r=0.52, p<0.01). All other parameters (including the differences between phase IV and baseline) were not correlated to absolute PTT at rest, during phase IV or PTT difference between both time points.

### Comparison Between Normative Data and Patients Groups

In a group analysis, patients with polyneuropathy showed reduced heart rate variability. Using an ANCOVA model adjusted for age, parameters obtained for these PNP patients during deep respiration and the coefficient of variation at rest were significantly lower than those from the control subjects. The Valsalva ratio and RMSSD at rest failed to distinguish between PNP patients and control subjects. Highest F-scores were found with the coefficient of variation both during deep respiration and at rest (Table **3**).

On an individual basis, 8 of 34 patients fulfilled the criteria of “pathological HRV”. This means they had more than 2 of 6 HRV parameters in the pathological range. Blood pressure regulation, as assessed by PTT reduction in phase IV of the VM, was also reduced in the PNP patients group (F=5.0, p<0.03). Individual analysis, however, revealed that only 3 of 34 patients were below the lower limit of normal values (PWL baseline – PWL phase IV < 7.7).

Heart rate variability was far less reduced in patients suffering from extrapyramidal disorders than in PNP patients. Just two parameters, the coefficient of variation during deep respiration (F=4.703, p<0.04) and the coefficient of variation at rest (F=4.128, p<0.05), were reduced. The remaining parameters were not different from control values. On an individual basis only 1 of 22 patients had to be classified as having “pathological HRV”.

Blood pressure regulation during phase IV of VM, however, was significantly impaired (F=10.074, p<0.005). Accordingly 5/22 patients had parameters below the lower limits of the normal range.

Direct comparison of both patient groups revealed that blood pressure regulation was significantly more impaired in patients with Parkinson syndromes (F=5.446, p<0.03).

### Sympathetic Skin Response (SSR)

The SSR on the feet was not obtained from the control group but only from patients. In 6 / 34 neuropathy patients, SSR could not be obtained, five of them had “pathological HRV”. SSR could not be elicited in 5 / 22 Parkinson patients, but none of them had “pathological HRV”.

## DISCUSSION

Our results suggest that the FAN ® system (Schwarzer, Germany) might be well suited to comprehensively assess autonomic function. We were able to define and apply to different patients groups normal values for heart rate variability (HRV) and for pulse wave transit time (PTT) changes during Valsalva manoeuvre. In particular the latter result could simplify the assessment of blood pressure regulation in the clinical routine.

### Reasonable Parameters to Assess HRV- the Normal Data

In a first step we defined lower limits of normal HRV investigating 190 normal subjects. All single parameters followed a logarithmic decrease with age. This is in accordance with previous studies [23]. Other biological markers had no significant effect on HRV. Each parameter of HRV was plotted against age and the lower limit of normal was defined as the 5%-tile of these data. This limit is moderately rigorous when compared to other studies [24]. The posture index and the max/min ratio during deep respiration had too huge a variability to define reasonable lower limits of normal. The posture index is further prone to movement artefacts and difficult to assess in, for example, bedridden patients [17]. Both parameters are therefore dispensable. For clinical routine, HRV parameters can be classified into three age groups (below 40, 40 to 60, 60 or older) [25]. Using this classification 96% of our normal subjects had 2 or less of the remaining 6 parameters of HRV in the pathological range. The assessment of these 6 parameters is necessary because one parameter alone would obviously not be precise enough to diagnose cardiac autonomic failure, otherwise, these 25% of healthy subjects would have been misclassified. HRV is an indirect measure and may be influenced by blood volume, baroreceptor activity or vasoconstriction [26, 27]. This might be the reason why test-retest correlation was moderate. In particular when active participation is required, control of confounding variables seemed difficult [28-30].

The decrease of HRV *in the time domain *with age and the lack of sex effects were in good accordance with previous reports about normative date on HRV in normal subjects. [37, 38].* In these studies s*pectral analysis has been also used to study autonomic nervous system function, in particular for differentiation between parasyspathetic and sympathetic function. Since specificity of spectral HRV components has been questioned several times [39] we intentionally focused on the better defined time domain analysis in the present study. The FAN®, nevertheless provides quantitative data of Fast Fourier Transformation in the low (0.01-0.05 Hz), medium (0.05-0.15 Hz) and high (0.15-0.50 Hz) frequency band.

### PTT as a Surrogate for Blood Pressure

For quantitative evaluation of blood pressure, continuous measurement is a prerequisite. However, continuous blood pressure measurement normally requires expensive, sophisticated hardware [31]. PTT, the time lag between ECG and the physical pulse wave, has been shown in previous studies to be negatively correlated to systolic blood pressure during resting conditions, correlations to diastolic blood pressure and blood pressure changes during cold pressor test were weak [32, 33]. This is exactly what we found. While in resting periods there was a linear relationship between PTT and blood pressure, the correlation became obviously much more complicated if blood pressure dynamically changed. One possibility to increase the coherence between blood pressure and PTT would be to subtract pre-ejection period (the R-wave/mechanical cardiac delay) for calculation of PTT [34]. Pre-ejection period has been shown to account for about 12-35% of the variability of PTT to the fingers [33]. This could in particular increase the correlation between diastolic and mean blood pressure and PTT. Calculating the pre-ejection period, however, requires either cardiac bioimpedance measurement [33] or phonocardiography [20]. This would level the major advantage of PTT – the ease of assessing. Although there are no systematic investigations so far it seems obvious that another possibility to reduce the influence of the pre-ejection period would be to vary the site of pulse wave recording. We measured the pulse wave at the earlobe leading to a relatively short PTT (154 ms) and possibly a relatively big pre-ejection period effect. Recording at the toe increases PTT to about 400 ms. Under these circumstances the pre-ejection effect might be minimized and correlation between PTT and arterial pressure could be enhanced [21].

However, we never intended to introduce PTT as a substitute of absolute blood pressure recording. The PTT in our study must be regarded as a relative parameter. It allows only the estimation of blood pressure changes. Our results show that if this prerequisite is considered, PTT might indicate cardiovascular changes during VM as it has been shown before in hemodialysis [20] and spinal anesthesia [21]. PTT during VM shows an inverse picture of the well-known blood pressure response [35]. That is, there was PWL reduction during phase IV of the VM. We focussed on phase IV, since the evaluation routine of the FAN defines VM phases on a time-basis. Phase IIb is less reliably defined due to individual differences [36]. We have been able to define lower limits of normal for phase IV and even test-retest reliability was fairly good within one session.

### The Significance of the Normal Values

In order to get an impression whether our normal data might be sensitive enough to detect autonomic disturbances we investigated patients with polyneuropathy and Parkinson syndromes. Polyneuropathy is associated with “cholinergic” autonomic failure - reduced HRV and absent SSR [14]. In our study eight patients had to be classified as “pathological HRV” and 6 of them also had absent SSR . This is a reasonable number in an unselected population of neuropathy patients. Parkinson patients predominantly suffer from orthostatic hypotension (“adrenenergic”) rather than from impairment of “cholinergic” function [2]. Accordingly Parkinson patients had predominant restriction of blood pressure regulation in our study.

We appreciate that our patients’ groups are inhomogeneous (neuropathy) and under medication (Parkinson’s disease). However, the aim of investigating patients in addition to normal subjects has been to provid an estimate about the value of our normal data to prove “normality”. This has been accomplished. For such a goal the cause of autonomic failure seems of minor importance.It was astonishing that we were able to detect exactly the pattern of autonomic failure which could be expected from previous studies.

In conclusion our study provides data showing that the FAN® might be a useful tool to assess different function parameters of the autonomic nervous system. In particular the assessment of PTT offers the opportunity to investigate large numbers of patients with sympathetic failure.

## Figures and Tables

**Fig. (5):**
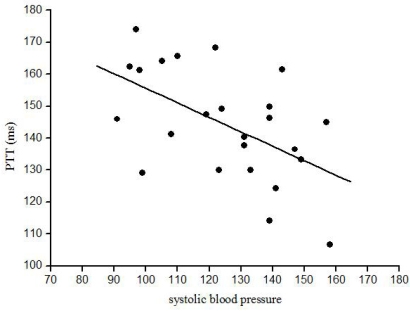
PTT at rest was significantly correlated to systolic blood pressure (r=-0.59, p<0.005).

**Table 1.** Listed are the Lower Limits of Normal for Each Parameter of HRV Corresponding to the Three Different Age Groups. Max/min During Deep Respiration and the Posture Index, which do not Significantly Contribute to Normal Data, are Marked in Grey

Age	n	DR: VC	DR: RMSSD	DR: max-min	VR	Rest: VC	Rest: RMSSD	DR: max/minn	PI
<40	96	4.63	21.03	87.20	1.36	3.57	16.27	1.11	1.06
40-60	71	3.10	12.88	46.00	1.19	2.37	11.29	1.05	1.06
>60	23	2.13	7.24	25.40	1.13	1.40	8.97	1.03	1.03

DR = deep respiration; VM = valsalva manoeuvre; Rest = HRV at rest; PI = posture index , VC = coefficient of variation; RMSSD = root mean square of successive differences.

**Table 2.** Statistical Evaluation of Test- Retest Correlation (n=10). Max/min During Deep Respiration and the Posture Index, which do not Significantly Contribute to Normal Data, are Marked in Grey

Statistical value	DR: VC	DR: RMSSD	DR:max-min	VR	Rest:VC	Rest: RMSSD	DR: max/min	PI
Pearson r	0.794	0.697	0.709	0.248	0.188	0.709	0.736	0.529
p-value	<0.01	<0.03	<0.03	n.s.	n.s.	<0.03	<0.02	n.s.

DR = deep respiration; VM = valsava manoeuvre; Rest = HRV at rest; PI = posture index , VC = coefficient of variation; RMSSD = root mean square of successive differences.

**Table 3.** Comparison Between Normal Subjects and Patients with Polyneuropathy. HRV was Significantly Reduced in the Patient Group. Max/min During Deep Respiration and the Posture Index, which do not Significantly Contribute to Normal Data, are Marked in grey

Statistical value	DR: VC	DR: RMSSD	DR: max-min	VR	Rest: VC	Rest: RMSSD	DR: max/min	PI
Ancova F	10.854	8.492	9.016	0.380	6.512	0.493	6.467	2.70
p-value	<0.01	<0.04	<0.005	n.s.	<0.02	n.s.	<0.02	n.s.

DR = deep respiration; VM = valsava manoeuvre; Rest = HRV at rest; PI = posture index , VC = coefficient of variation; RMSSD = root mean square of successive differences.
